# Key Molecular Events in Cervical Cancer Development

**DOI:** 10.3390/medicina55070384

**Published:** 2019-07-17

**Authors:** Shandra Devi Balasubramaniam, Venugopal Balakrishnan, Chern Ein Oon, Gurjeet Kaur

**Affiliations:** Institute for Research in Molecular Medicine, Universiti Sains Malaysia, 11800 Minden, Pulau Pinang, Malaysia

**Keywords:** cervical cancer, cervical intraepithelial neoplasia, human papillomavirus, carcinogenesis, viral oncoprotein, tumour suppressor gene

## Abstract

Cervical cancer is the fourth most common cancer among women. Infection by high-risk human papillomavirus (HPV) is the main aetiology for the development of cervical cancer. Infection by high-risk human papillomavirus (HPV) and the integration of the HPV genome into the host chromosome of cervical epithelial cells are key early events in the neoplastic progression of cervical lesions. The viral oncoproteins, mainly E6 and E7, are responsible for the initial changes in epithelial cells. The viral proteins inactivate two main tumour suppressor proteins, p53, and retinoblastoma (pRb). Inactivation of these host proteins disrupts both the DNA repair mechanisms and apoptosis, leading to rapid cell proliferation. Multiple genes involved in DNA repair, cell proliferation, growth factor activity, angiogenesis, as well as mitogenesis genes become highly expressed in cervical intraepithelial neoplasia (CIN) and cancer. This genomic instability encourages HPV-infected cells to progress towards invasive carcinoma. The key molecular events involved in cervical carcinogenesis will be discussed in this review.

## 1. Introduction

Cervical cancer is the fourth most common cancer among women worldwide, with an estimated 570,000 cases and 311,000 deaths, reported in 2018 alone [1]. It has been estimated that about 85% of worldwide deaths from cervical cancer occur in low and middle-income countries [2], where the death rate is 18 times higher than in developed countries [3]. The incidence death rates are very high in some of the countries of Sub Saharan Africa, Latin America and Asia [4]. Several studies have reported that low economic status, poor personal and sexual hygiene, smoking, early age of sexual activity and having multiple sexual partners are some of the risk factors for the development of cervical cancer [3,5]. Human papillomavirus is the main aetiological factor in the process of carcinogenesis [6]. However, not all human papillomavirus (HPV) infections suffered by women culminate in cervical cancer. High-risk HPV genotypes trigger the progression of a normal cell into a precancerous lesion and later into an invasive lesion. The pathogenesis of HPV infection involves the overexpression of viral oncoproteins that can inhibit a variety of cellular proteins and affect biological processes including cell proliferation, cell cycle, and apoptosis. The viral and host cellular alterations that induce cervical carcinogenesis provide deep insight into the nature of the disease as well as inspire the development of specific molecular targeted therapy.

## 2. Pathological Changes in the Cervix

**Figure 1:**
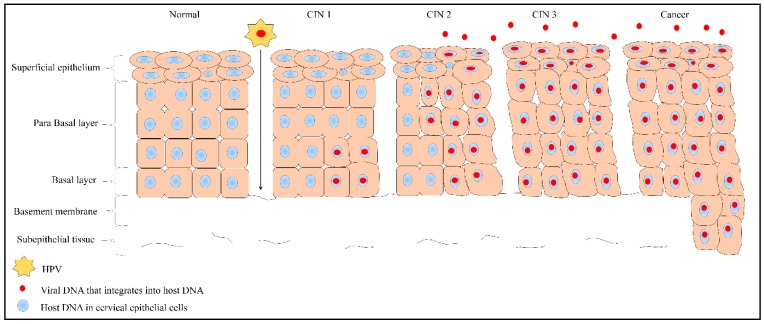
Distribution of normal and human papillomavirus (HPV)-infected squamous epithelial cells in normal, precancerous lesions (mild, moderate and severe dysplasia; CIN 1, CIN 2, and CIN 3, respectively) and cancer of the cervix. The initial stage of carcinogenesis is controlled by viral HPV integration and host factors. HPV enters the basal epithelial cells through a micro-wound. Subsequently, the virus integrates its genome into the host genome in the nucleus through nuclear envelope breaks. Once it enters the nucleus, HPV takes over control of the host genome, self-replicates, and spreads throughout the epithelium. Further replication of the viral genome causes the host cells to grow irregularly and in a disorganized manner compared to normal cells. Subsequently, the virions are sloughed off with the dead squamous cells of the host epithelium, facilitating further transmission.

The cervix is the lower part of the uterus; it is cylindrical in shape and is connected to the vagina through the endocervical canal [7]. The endocervical canal is lined with stratified squamous epithelium and columnar epithelium that cover the ectocervix and endocervix, respectively. The transition zone between these cells is called the squamocolumnar junction. Any premalignant transformation of cells occurs mostly at the squamocolumnar junction and is closely associated with the predominantly genotype 16 and 18 high-risk HPV. The premalignant changes or dysplasia of squamous cells in the cervical epithelium are collectively known as cervical intraepithelial neoplasia (CIN). CIN can progress to carcinoma in situ and invasive carcinoma if it is not treated at an early stage or if the HPV is able to deactivate the host cellular functions [3]. Moreover, there is strong evidence that certain viral proteins in HPV are responsible for dysplastic changes in infected cells and these too, cause transformation of precancerous to cancerous lesions [8]. A person who is diagnosed with mild dysplasia or CIN 1 (low-grade CIN) might recover from the infection with the aid of the host’s immune system [9]. Histologically, CINs are graded according to severity [8]. Figure 1 shows the distribution of epithelial cells in the normal cervix and HPV-infected cells in precancerous (CIN 1, CIN 2 and CIN 3) and cancerous conditions. The epithelial cells are well organized in a normal cervix. However, in CIN and cancer, the cells infected with HPV become dysplastic. CIN 1, also known as low-grade CIN (LGCIN) denotes mild dysplasia where the lower one-third of the epithelium shows dysplasia [8,10,11]. When two-thirds of the epithelium is affected, it is referred to as CIN 2 or moderate dysplasia [8,12]. Severe dysplasia (CIN 3) is graded when more than two thirds of the full thickness of the epithelium is affected [8,13]. CIN 2 and CIN 3 lesions are collectively classified as high-grade CIN (HGCIN) [10,11].

## 3. Human Papillomavirus 

Human papillomaviruses (HPV) are small non-enveloped double-stranded DNA viruses with genomes containing 8 kb of DNA sequences. To date, there have been in excess of 200 HPV genotypes identified and these are classified into mucosal and cutaneous HPV [14]. The high-risk HPVs are associated with mucosal infection and the low-risk HPVs are associated with cutaneous lesions. Low-risk HPV types such as HPV 6, 11, 42, 43 and 44 are related with benign lesions that often form warts but are rarely found in malignant tumours [15]. Conversely, the high-risk HPVs such as HPV 16, 18, 31, 33, 34, 35, 39, 45, 51, 52, 56, 58, 59, 66, 68 and 70 are associated with cervical cancer lesions with the most prevalent high-risk HPV being HPV 16 followed by HPV 18 [16]. In the event of carcinogenesis, 70% of cervical cancers and 50% of CIN 3 lesions develop because of persistent infection of either HPV 16 or HPV 18 [17]. The HPV genome codes for only eight proteins, which are listed in Table 1, with each playing a major role in the HPV life cycle and the transformation of host cells into cancerous cells [18]. Following viral infiltration of the basal epithelial cells, the aforementioned viral particles are released to facilitate the integration of the viral genome into the host’s by damaging several cellular pathways in the host cells. Table 1 lists the major functions of the HPV viral proteins in initiation of infection and subsequent progression to cancer.

## 4. Key Factors in Initiation of Cervical Cancer 

Progression of HPV infected epithelial cells to invasive cancer is a long-term process associated with the accumulation of DNA alterations in host cell genes. These alterations involve both epigenetic and genetic changes in oncogenes and tumour suppressor genes. HPV enters the host basal squamous cells through a micro-wound or abrasion. The virus must be able to integrate into the host cell to initiate the infection, where a series of genetic events occur within the basal epithelial cells directly enabling viral replication. These events then establish an environment permissive for neoplastic progression [19]. At this point, the virus must evade the host immune system to ensure its continuous replication in the basal epithelial cells. CIN 1 is the stage when the virus persistently infects the cervical cells. The transition from a precancerous lesion to invasive carcinoma takes at least 10–12 years [20,21]. CIN 1 lesions that do not regress may develop into CIN 2/3 within 2–3 years following infection [22].

In general, upon infection, the host cell activates the innate and adaptive immune systems, which are controlled by the major histocompatibility complex (MHC) class I and II. MHC class I molecules present antigen to cytotoxic T cells with CD8+ receptors whereas MHC class II molecules present antigens to helper T cells with CD4+ receptors. During HPV infection, the virus enters the epithelium, which is the outermost physical barrier against infections, and activates the host innate immune system. The macrophages, Langerhans cells, and the natural killer cells try to inhibit HPV by expressing toll-like receptor (TLRs) [23]. TLRs recognize the viral components and activate transcription factor-like nuclear factor kappa B (NF-κB) and interferon response factor-3 (IRF3) to produce cytokines [24]. In this scenario, TLRs also indirectly activate MHC class I and class II. It is reported that the viral protein E5, expressed by high-risk HPV, can inhibit MHC class I [25]. In addition, the E6 viral oncoprotein is able to inhibit the TLR which then activates IRF3 [26].

The toll-like receptors (TLRs) in the host cells play a fundamental role in pathogen recognition and activation of innate immunity and induce production of cytokines necessary for the development of effective immunity. It has been established that TLR 3, 4, 7, 8, and 9 play a major role in antiviral immunity by triggering the downstream production of interferons (IFNs) [27]. During the early stage of cervical carcinogenesis, the cervical epithelium, which is undergoing differentiation, triggers the regulation of TLR receptors, which in turn elicits antiviral responses via IFN-regulatory factor (IRF) [28]. A study showed that expression of TLR-9 gene differs between stages of cervical cancer development. It was reported that the TLR-9 gene showed lower expression in CIN 1 compared to CIN 2/3 and displayed the highest expression in squamous cell carcinoma (SCC) group samples [29]. However, continuous overexpression of E6 and E7 oncoproteins may down-regulate TLR-9 and the ensuing interferon response is impaired, resulting in evasion of immune response leading to persistent infection [30]. It is reported that the HPV E7 oncoprotein is able to bind to Histone deacetylase 1 (HDAC1) and prevent acetylation of histones, thereby deregulating TLR9 signalling [31]. In another study, it was reported that HPV upregulates epidermal growth factor receptor (EGFR) to drive interferon-related developmental regulator 1 (IFRD1) expression into decreasing cytokine production by suppressing NF- κB [32].

HPV can escape the innate immune system and integrate into the epithelial cells. Upon entering the cell, the dendritic cells engulf the HPV antigen and undergo a maturation process. The phagolysosome then sends the antigen to bind on MHC class I molecules cell surface or II. Upon binding, the CD4+ and T cells will also bind to the T-cell receptor (TCR). The antigen presenting cells (APC) will then activate CD4+ and T cells to show cytotoxic effect. Activation of APC will activate the pro-inflammatory and antiviral cytokines such as IFN-γ and tumour necrosis factor alpha (TNF-α). This leads to stimulation of macrophages and promotes inflammation or tumour immunity. Moreover, the interleukins are also stimulated to respond to extracellular pathogens. However, activation of APC also triggers the production of Tregs (regulatory T cells). Tregs will activate interleukin (IL-10) and transforming growth factor beta (TGF-β), which will inhibit the function of APC. Therefore, in HPV cancer progression, the transformation of cells from normal to precancerous lesions and to cancer is associated with the amount of Treg cells being produced. It is reported that women with persistent HPV 16 infection have been observed to have significantly higher Tregs than HPV-negative women [33]. Moreover, in another study, Treg-inducing molecules like TGF-β1 have been shown to be increased in lesions progressing from CIN 1 to invasive cervical cancer [34]. 

**Figure 2:**
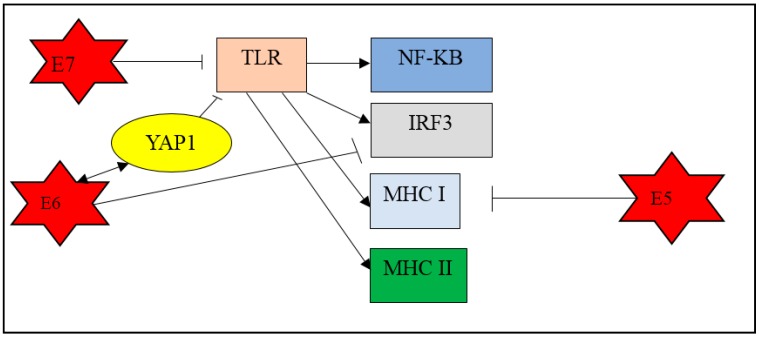
Mechanisms involved upon HPV infection. Upon HPV infection, the host immune system triggers the toll-like receptors (TLR) to activate the nuclear factor-kappa B (NF-κB) and interferon regulatory factor 3 (IRF3) to activate pro-inflammatory factors and antiviral cytokines. The TLR also activate the major histocompatibility complex (MHC) class I and II. However, the HPV can express its viral oncoprotein E5 to inhibit the MHC class I mechanisms. The oncoprotein E6 has the ability to inhibit the production of IRF3. The oncoprotein E6 also binds to Yes-associated protein (YAP1), preventing degradation of YAP1, and inhibiting the TLR.

**Figure 3:**
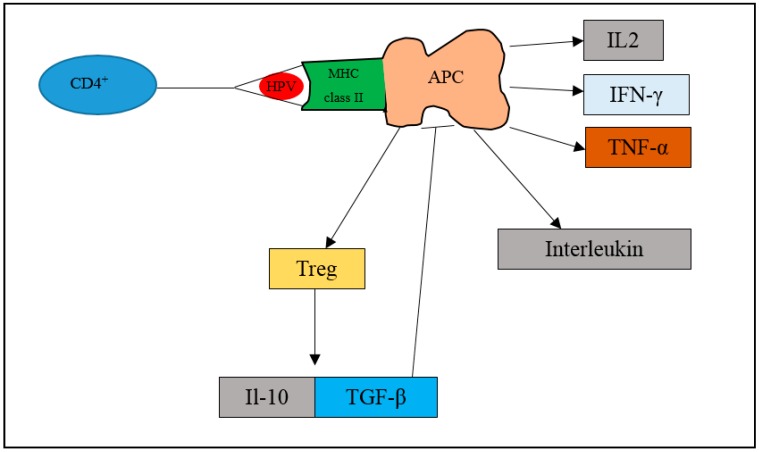
Mechanisms involving MHC class II upon HPV infection. When HPV enters the epithelium, the host immune system identifies the HPV antigen and phagocytosis is initiated. The phagolysosome sends the antigen to bind on MHC class II molecule. The antigen presenting cell (APC) will then activate CD4+ and T cells to exert cytotoxic effect. Activation of APC will activate pro-inflammatory and antiviral cytokines such as interferon gamma (IFN-γ), tumour necrosis factor alpha (TNF-α), and interleukin 2 (IL-2). The interleukins are also stimulated. Activation of antigen presenting cells (APC) also triggers the production of regulatory T cells (Tregs). Tregs will activate interleukin 10 (IL-10) and transforming growth factor beta (TGF-β), which will inhibit the function of APC.

A study by Gius established that at an early stage of infection, HPV alters the cellular immune system IL receptor 1 (IFNAR1), epithelial membrane protein 1 (EMP1), and interleukin 1 receptor antagonist (IL1RN) genes, suggesting that at early stage of HPV infection, the virus alters the expression of these genes so as to evade the host immune system and permits the progression of the infected cells towards becoming cancerous [19]. In addition, another researcher found that interleukin 1 receptor type 2 (IL1R2) is downregulated during the progression of cervical cancer [35]. A recent study reported that the Hippo-Yap pathway plays a role in development of cervical cancer [36]. The major effector of the Hippo signalling pathway, the Yes-associated protein (YAP1), interacts with the HPV E6 oncoprotein to initiate and promote the progression of cervical cancer. The HPV oncoprotein binds to YAP1 and works synergistically to prevent degradation of YAP1 [37]. Amplification of the oncogene YAP1 was demonstrated in human squamous cervical cancers [38], while its overexpression in cervical epithelial cells induced the development of squamous cell carcinoma in a mouse model [36]. Moreover, hyperactivated YAP1 increased the susceptibility of HPV infection by upregulating the putative HPV receptors, comprising EGFR, integrin receptor subunit alpha 6 (ITGRA6) and syndecan 1 (SDC1) [36,39]. The study also demonstrated that the upregulation of YAP1 downregulates TLR 2 and 4, which are key components in innate immunity [36]. YAP1 negatively regulates the production of type I interferon by suppressing TANK-binding kinase 1 (TBK1) activity [40]. TBK1 is a molecule that is activated by pattern recognition receptors (PRRs) upon viral infection, an important protein involved in signalling pathways such as cell proliferation, autophagy and insulin signalling pathways [41]. The specific mechanisms associated withYAP1-induced cervical cancer are still being investigated, although YAP1 could be a potential prognostic biomarker in cervical cancer [39]. In summary, HPV-associated cervical cancer develops upon evasion of the host’s immune system, leading to further host cellular dysfunction. Figure 2 and Figure 3 show the key events that occur when the expression of HPV E6 and E7 oncoproteins leads to evasion of the host’s immune system.

## 5. Progression of Precancerous Lesions to Cancer 

Carcinogenesis is a process by which normal cells become abnormal and transform into cancer cells. Infection by high-risk HPVs leads to the development of precancerous lesions in the cervix. Oncogenesis only occurs as a result of prolific genomic and epigenomic alterations in these cells. HPV infection alone is insufficient to trigger the development of cervical cancer. There are several other factors involved during the incubation period of the virus in the host tissue.

The HPV oncoproteins, mainly E6 and E7, play a major role in the alteration of host cellular function. HPVs overexpress E6 and E7 oncoproteins to disrupt the normal function of tumour suppressor genes in the host. Upon integration, the viral proteins begin to damage the host cells. Host cells have evolved a special mechanism to repair the damage inflicted upon their DNA via DNA damage response. Only once the damaged DNA is repaired can the cell cycle checkpoints be mitigated, and the cells be allowed to continue dividing. Under certain circumstances, if the cells are unable to repair the DNA damage, then apoptosis takes place. However, in HPV-related cancer, the E6/E7 viral proteins disrupt cell cycle checkpoint control by both inhibiting cyclin dependent kinase (CDK) inhibitors (p21, p27, p16) and degrading p53 and retinoblastoma (pRb) [42]. Degradation of p53 by E6 oncoprotein-induced apoptosis allows cells to continue replicating. HPV takes advantage of this damage response pathway for its own replication and produces ample numbers of episomal HPV, which are needed for HPV DNA to integrate into the host genome. Thus, degradation of pRb by E7 oncoprotein will cause unscheduled entry into S phase of the cell cycle [43] which eventually promotes cells to proliferate. HPV viral replication requires the cell to enter the S phase of cell cycle. This is achieved by inactivating pRb and releasing the transcription factor (E2F) family transcription factors that allow progression of the cell cycle from the G_1_ checkpoint. The E2F family can mediate both cell proliferation and p53-dependent or independent apoptosis. Overexpression of E2F leads to both the inhibition of cyclin D1, dependent kinase activity and the induction of the CDKN2A, cyclin-dependent kinase inhibitor 2A (p16) gene product overexpression.

The main reason for HPV to disrupt the host gene is so that it can continue to replicate and survive in the host tissue by exploiting the host cell cycle machinery. It is understood that the development of cervical cancer is a long process from normal to CIN 1, then CIN 2/3 and ultimately into cancer. Although the key regulators for cervical cancer are the HPV oncoproteins, there are significant genomic alterations involved in the transformation from a precancerous to a cancerous state. In the transition from CIN 1 to CIN 2/3 there is an accumulation of abnormal cells in up to two thirds of the thickness of the epithelium. This is achieved through cell proliferation, a critical factor in tumour development.

According to Gius et al., at the early stage of HPV infection, proliferative genomic signatures are significantly involved [19]. The study found upregulation of CDKN2A, Kinesin family member (KIF23), Centromere protein E (CENPE), and Integrin subunit alpha V (ITGAV) genes that are involved in cell cycle regulation and promotion of cell proliferation [19]. Moreover, in a study on CIN 2/3, it was found that genes involved in cell division, DNA replication, the cell cycle, and transcription regulation were highly upregulated, these included alpha actinins (BUB1B), mitotic arrest-deficient 2 (MAD2L1), checkpoint kinase 1 (CHEK1), cyclin B1 (CCNB1), cyclin B2 (CCNB2), cell division cycle 20 (CDC20), cell division cycle 6 (CDC6), cyclin A2 (CCNA2), replication factor 3 (RFC3), replication factor 4 (RFC4), focal adhesion kinase (FEN1), and proliferating cell nuclear antigen (PCNA) genes [44]. Furthermore, Niu et al. found that cell proliferation genes were highly upregulated in CIN 2/3 when compared to a normal cervix [35]. A healthy cell has a perfect balance between cell proliferation and cell death. An imbalance can lead to diseases including cancer. Hence, we postulate that, during CIN 2/3 stage, the cells rapidly undergo the cell proliferation process. This is the most crucial stage in the progression from healthy cells to cancerous ones. In fact, apoptotic activity is downregulated in precancerous and cancer cells, thus promoting cell survival and cell proliferation [45]. There are multiple genes involved in cell proliferation, as reported in several studies. However, most studies focus on p16 also known as CDKN2A. Several studies suggested CDKN2A is a potential biomarker in cervical cancer. When the cells rapidly proliferate and replicate in the CIN 2/3 stage, they may lack nutrients due to the high metabolic rate during cell division. A study suggested that these cells activate the neovascularization pathway [19] to ensure an adequate supply of nutrients during the development of cancer cells. Angiogenesis occurs in the precancerous stage as an indication of development into cancer [46].

**Figure 4:**
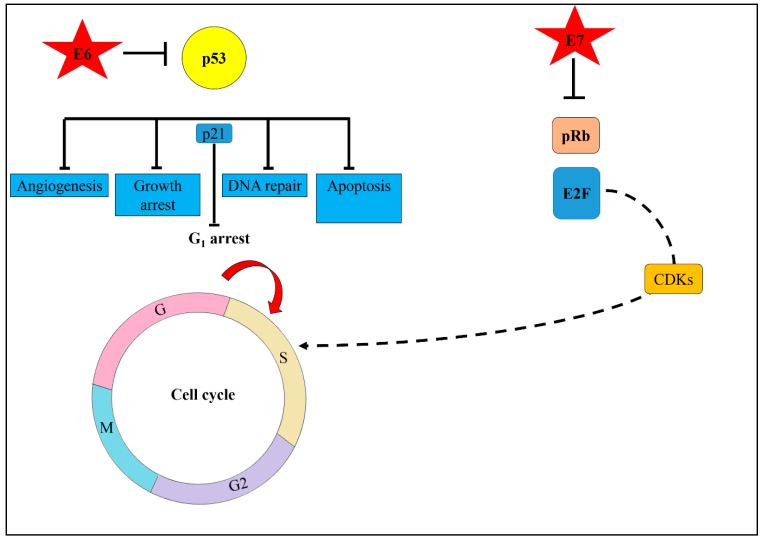
Schematic diagram illustrating the role of HPV oncoproteins in cervical carcinogenesis. Upon infection, the viral oncoprotein E6 binds to p53, a tumour suppressor protein and disables its normal function. The host cell’s ability to undergo DNA repair, apoptosis, or growth arrest and angiogenesis is disrupted. Activation of p53 will activate cyclin-dependent kinase inhibitor (p21) to force cells to remain at G_1_ arrest. However, upon HPV infection, the E6 degrades p53 which causes cells to enter S phase of cell cycle. Simultaneously, the E7 oncoprotein binds to retinoblastoma (pRb). The binding of E7 to pRb causes it to release E2F, a transcription factor that activates the cyclin dependent kinase (CDKs). This causes the cell cycle to lose control, allowing the cells to re-enter into S phase of the cell cycle. When infected cells differentiate and proliferate at a high level, the development of abnormal dysplastic cells will be promoted.

Angiogenesis is the formation of new blood vessels which are essential for tumour growth and metastasis formation. The vascular endothelial growth factor (VEGF) is the key stimulator for angiogenesis. Activation of VEGF triggers proliferation, differentiation, and migration of endothelial cells. Moreover, VEGF also increases vascular permeability and enhances production of proteases, which are involved in alteration of the extracellular matrix process. Thomas et al. also found that epidermal growth factor receptor (ERBB) genes are upregulated in the precancerous stage. In cancer development, enhanced production of ERBB genes promote cell proliferation and inhibit cell apoptosis [46]. In summary, the cells dysregulate several cellular functions to promote cell proliferation during the transition from precancerous to cancer. Figure 4 shows key molecular events triggered by HPV oncoproteins during the process of cervical carcinogenesis.

## 6. Genomic Alterations in Cancerous Stage

As the disease progresses to invasive carcinoma, a significant difference in the gene expression arises, and the number of genes deregulated increases with progressive pre-neoplastic conditions [19,46,47]. Failure to inhibit cell proliferation is the crucial element in the development of cancer. These cells maintain their hyperproliferative state by upregulating genes that control multiple steps of DNA replication [48]. These genes include the minichromosome maintenance (MCM) proteins; MCM2, MCM4, MCM5, MCM6, and MCM10 which are all important proteins in replication initiation and allow for DNA polymerase to initiate DNA replication [49]. Studies have proven that MCM is highly upregulated in cervical cancer [46,48] and is being investigated as a biomarker for the screening of cervical lesions [50]. When these genes are activated, the genes coding for DNA polymerase; polymerase epsilon catalytic subunits POLE2 and POLE3 are concordantly upregulated as they need to work together with MCMs to initiate the mitotic process [48]. Proliferating cell nuclear antigen (PCNA) is most relevant in cell proliferation and is involved in the radiation-repair genes (RAD6)-dependent DNA repair pathway [51]. Several studies using immunohistochemistry and microarray found that expression of PCNA increased as the disease progressed [46,52,53,54].

Furthermore, to accomplish cell proliferation activity, the genes involved in mitosis also play a significant role. The formation of the mitotic spindle is important and is controlled by the cell cycle, which appears during the G_1_ to S phase and disappears after cytokinesis [55]. The microtubule nucleation factor (TPX2), also known as restrictedly expressed proliferation association protein (REPP86), is a microtubule protein which is associated with spindle development. An increased expression of this gene in tumours results in abnormal centrosome amplification, aneuploidy formation and malignant transformation of cells. Besides promoting proliferation, it is also involved in cell cycle and apoptosis regulation, tumour differentiation, metastasis, and recurrence. In cervical cancer, TPX2 is highly expressed in squamous cell carcinoma and increases with the tumour grade [56,57]. The gene is considered a risk factor for metastasis of cervical cancer and is also a potential biomarker for cervical cancer diagnostics. In addition to mitosis, it was found that genes involved in the mitotic spindle checkpoint Bub1 which monitors the assembly of the mitotic spindle and ensures the accurate segregation of sister chromatids, are highly expressed [58].

The activation of the cell cycle is a factor associated in HPV-associated cervical cancer. It has been reported that the cyclin proteins CCNA2 and CCNB1 and their associated kinases CHEK1 and CDK1 were significantly upregulated in cervical cancer tissue; these proteins promote cell cycle transition from the G_1_ to the S phase and from the G_2_ to the M phase [48]. PCNA was also found to be upregulated in cervical cancer tissues [48]. CDC20 is upregulated in both CIN 2/3 and SCC of the uterine cervix [59]. Replication factor C (RFC) is important for DNA replication and cell cycle control [60]. Another study found that RFC3 and RFC4 promoted tumour cell proliferation, and the high expression of RFC3 was associated with poor prognosis in a variety of cancers [61,62]. 

In the invasive carcinoma stage, genes related to cell cycle regulation and metastasis are highly expressed [46]. The majority of studies are in concordance with Gius et al., who reported that during transition from CIN 2/3 to cancer, there is a predominance of cellular stress due to cellular overcrowding causing activation of genes that trigger angiogenesis and invasion [19]. The cells need to overcome the physical barrier of the epithelial cells and basement membrane to invade deeper into the tissue. In addition to this, more nutrients are necessary for cell expansion. Another study discovered that during the transition into cancerous cells, biological processes such as extracellular matrix (ECM) organization, epithelial cell differentiation, and collagen fibril organization genes were involved [44]. Among the identified genes were phosphoinositide-3-kinase (PIK3CA), vascular-endothelial growth factor A (VEGFA), integrin subunit alpha 1 (ITGA1), protein tyrosine kinase (PTK2), integrin subunit beta 1 (ITGB1), alpha actinins (ACTN1), fibronectin 1 (FN1), collagen type 1 (COL1A1), collagen type 2 (COL1A2), and syndecan 2 (SDC2) that is associated with focal adhesion [44]. Focal adhesions are large macromolecular assemblies through which mechanical force and regulatory signals are transmitted between the extracellular matrix (ECM) and interacting cells. Focal adhesion kinase (FAK) is the key enzyme regulating the formation of focal adhesions, and is a key regulator of survival, proliferation, migration, and invasion, this activity endows cells with higher motility [63]. Indeed, FAK overexpression has been identified in aggressive cervical cancer [64]. Aberrant expressions of multiple genes are involved in the progression to cervical cancer, whereby cells acquire the ability to sustain proliferation and resist attempts at cellular death or apoptosis. Furthermore, these abnormal cells acquire the ability to invade underlying tissues.

## 7. Potential Biomarkers in Cervical Cancer

The knowledge of specific molecular mechanisms underlying the etiopathogenesis of cervical cancer is important in the discovery of potential molecular markers. To date, several lines of evidence support the prospect of biomarkers in the identification of precancerous lesions and early stage cervical cancer, which will promote early treatment and provide a better prognosis to individuals. Upregulation of CDKN2A (p16) in early cancer is an indication of the host’s response in deactivating pRb and releasing the E2F family. The interaction between high-risk HPV and CDKN2A plays an important role in cervical carcinogenesis. The immunohistochemical expression of CDKN2A is higher in squamous cell carcinoma compared to other HPV-related tumour types [65]. Moreover, CDKN2A is associated with high-risk HPV infection and CIN 2/3, supporting its role as a useful biomarker for precancerous lesions and cervical cancer [19,35,66,67,68]

Another promising biomarker in cervical cancer is Ki-67, a nuclear antigen associated with cell proliferation. The protein is present during all the active phases of the cell cycle, (G_1_, S, G_2,_ and mitosis), but is absent in resting cells (G_0_); hence, it is used to determine the growth fraction of a cell population [69]. Several studies have shown that co-expression of p16 and Ki-67 improved diagnostic accuracy in cervical cancer screening and that Ki-67 expression increased in a linear manner with tumour grade [70,71,72]. In the normal cervix, Ki-67 is localised within the basal and suprabasal layers, whereas in CIN it is expressed throughout the epithelial layers, denoting cell proliferation [73]. Ki-67 is also a potential biomarker for CIN 1 [74].

Evasion of the host immune system is crucial in the development of cervical cancer. The HPV infection induces a broad-spectrum of host immune responses that comprise major pathways such as toll like receptors and NF-κB. Many proteins, including programmed death ligand (PDL1), importin-β, exportin-5, cellular-FLICE-like inhibitory protein (c-FLIP), myeloid leukaemia cell proliferation protein (Mcl1), have been proposed as novel biomarkers for CIN and cervical cancer [74]. However, more research is needed before they can be introduced into cervical cancer screening programmes.

## 8. Conclusions

HPV infection plays a major role in cervical carcinogenesis. The overexpression of E6/E7 oncoproteins is the key factor that affects tumour suppressor genes, mainly those regulating the cell cycle, which then alters many downstream pathways leading to cancer progression. Understanding the key molecular mechanisms perturbed in the progression from HPV-infected cells to cervical intraepithelial neoplasia and finally to invasive cancer provides an insight into the multitude of pathways involved and inspires the future development of targeted therapies.

## Figures and Tables

**Table 1:** List of human papillomavirus (HPV) viral proteins and their major functions.

Viral Protein	Protein Functions
**E1**	Viral DNA replication and transcription
**E2**	Viral DNA replication, apoptosis, transcription repressor of E6/E7
**E4**	Viral DNA replication
**E5**	Immune recognition (major histocompatibility complex, MHC)
**E6**	p53 degradation, alteration of cell cycle regulation, apoptosis resistance
**E7**	retinoblastoma (pRb) degradation, re-entry into S phase cell cycle, p16 overexpression
**L1**	Major viral capsid protein
**L2**	Minor viral capsid protein
